# A training curriculum for an mHealth supported peer counseling program to promote exclusive breastfeeding in rural India

**DOI:** 10.1186/s13006-023-00546-4

**Published:** 2023-01-19

**Authors:** Roopa M Bellad, Niranjana S Mahantshetti, Umesh S Charantimath, Tony Ma, Yukiko Washio, Vanessa L Short, Katie Chang, Parth Lalakia, Frances J Jaeger, Patricia J Kelly, Geetanjali Mungarwadi, Chandrashekar C Karadiguddi, Shivaprasad S Goudar, Richard J Derman

**Affiliations:** 1 KLE Academy of Higher Education and Research, Jawaharlal Nehru Medical College, Belagavi, Karnataka India; 2 Benten Technologies Inc, Manassas, Virginia USA; 3 Substance Use, Gender and Applied Research, RTI International, Research Triangle Park, North Carolina USA; 4 Thomas Jefferson University, Philadelphia, Pennsylvania USA

**Keywords:** mHealth application, Breastfeeding, Exclusive breastfeeding, Training curriculum, Peer counseling

## Abstract

**Background:**

Despite strong evidence about the benefits of exclusive breastfeeding, that is the baby receiving only breast milk, no other foods or liquids, rates have remained relatively unchanged over the past two decades in low- and middle-income countries. One strategy for increasing exclusive breastfeeding is through community-based programs that use peer counselors for education and support. The use of mobile health applications is also gaining increasing applicability in these countries. Minimal information is available about training peer counselors in the use of mobile technologies to support exclusive breastfeeding. The present article describes our curriculum in the state of Karnataka, India for supporting new mothers to exclusively breastfeed using a mobile health application in rural India.

**Methods:**

Twenty-five women from the community surrounding the city of Belgavi, Karnataka, India were trained to be peer counselors and to use a mobile health application to conduct a structured curriculum to support new mothers in exclusive breastfeeding. The three-day interactive training, conducted in March 2018, was based on the WHO breastfeeding course, translated, and adapted to the local culture The curriculum, which included information collected during a formative research process, consisted of eight visits, two during the antenatal period and continuing for six months postpartum. Twelve nursing and obstetric experts validated curriculum content. Pre-post-evaluation of the training focused on breastfeeding knowledge, self-efficacy, skills, and app usability.

**Results:**

We observed a significant increase in the mean scores for knowledge (*P* < 0.0001) and skills (*P* = 0.0006) from pre- to post-training. Age of the peer counselors and their own breastfeeding experience correlated significantly with the acquisition of knowledge and skills. The mobile health app showed high usability scores.

**Conclusions:**

The culturally adapted curriculum presented here, combined with an mHealth app, can be an important educational strategy for training rural women in the acquisition of exclusive breastfeeding knowledge and skills.

## Background

Exclusive breastfeeding (EBF), the practice of a baby receiving only breast milk, no other foods or liquids, significantly decreases mortality and morbidity in children less than five years of age, resulting in improved child survival and health [1, 2]. Exclusive breastfeeding can reduce infant mortality by preventing diarrhea and acute respiratory infections [3]. Increasing the rate of EBF in the first six months of life to at least 50 % is one of the six World Health Organization (WHO) global nutrition targets for 2025 [4].

Global rates of optimal breastfeeding practices, especially EBF, have remained stagnant over the past two decades in India and other low-and middle-income countries (LMICs), with only one in three infants under six months being exclusively breastfed [3]. The most recent India National Family Health Survey found that for the first six months of life, 56.4 % of infants in Karnataka, India were exclusively breastfed [5]. Achieving EBF requires a multi-pronged approach that involves both healthcare providers and policymakers, along with community participation and support [6, 7].

One strategy for increasing EBF is the use of community-based programs that use peer counselors (PCs) to educate and support mothers. Such programs have shown to be effective in increasing the initiation and duration of breastfeeding in diverse populations and settings, including LMICs. Successful PC programs have been shown to address a myriad of health-related problems, including HIV prevention, immunizations, alcohol use, and depression in several LMICs [8–18]. Effective training of PCs is a crucial prerequisite for the success of such programs.

Mobile health (mHealth) applications (apps) use mobile devices such as smartphones or handheld mobile tablets to enhance teaching, collaboration, and provision of medical care [19]. Such mHealth apps are increasingly popular to reach rural populations in LMICs with strong internet access capability [20–22], and is commonly available in India, mHealth-based programs have the potential to provide scalable public health interventions [23].

There is evidence that mHealth-supported community programs can improve the behaviors of Indian mothers, including the uptake of health services among pregnant and breastfeeding women with HIV [24]. A recent meta-analysis of studies conducted in six countries suggested that mHealth may be associated with improved maternal breastfeeding attitude, knowledge, initiation, and EBF duration [25]. Two additional reviews found evidence of positive results on EBF and other neonatal and maternal outcomes but stressed the need for both strong research methods and personalized contact [26]. It remains unknown, though, whether utilizing mHealth platforms to enhance PC efforts is effective at improving the infant feeding behaviors of mothers in India.

As part of a funded research project, *BEST4Baby*, we adapted the WHO’s breastfeeding curriculum to the local culture and trained rural mothers with prior breastfeeding experiences to serve as breastfeeding peer counselors. The training prepared them to counsel and support antepartum and postpartum mothers around the topic of EBF and infant feeding. To maximize the utility of the PCs, we designed the delivery of training content via a mHealth app on a Samsung Android tablet. The present article describes our curriculum in the state of Karnataka, India for supporting new mothers to exclusively breastfeed and documents pre-post changes of the peer counselors training curriculum.

### Development of the training curriculum

Using the results of qualitative research that we conducted through focus group discussions, we adapted the World Health Organization’s breastfeeding counseling course, and Haider and colleagues’ breastfeeding PCs training module to the cultural setting of the southern Karnataka state in the Kannada language [8, 27, 28]. Local breastfeeding practices, misconception and dangers of the use of top feeding were incorporated into the curriculum since the results of our formative research described mixed practices about prelacteal and supplemental feeding, reflecting older, traditional views. Names and figures in the module were based on how women looked and spoke in local communities. From the formative research, the involvement of the mother-in-law was added, as well as specifics about cultural practices such as the use of tim tim (herbal drops) and guti (locally made gruel mixtures). Research staff were experienced in the content area and are designated as national breastfeeding trainers in India; additional guidance was sought from twelve nursing and obstetric experts to develop the curriculum.

#### Training curriculum

A three-day PC training was conducted in the local language at the academic health center. Two days were dedicated to breastfeeding education, skills building, and the use of Best4Baby kit, such as a doll for positioning. The last day was dedicated to a half-day on the use of the mHealth app, with the remaining time for protocol training and skills assessment. Pedagogy across the three days included interactive lectures, demonstrations, brainstorming sessions, case-based learning, role-playing, and continued positive feedback to enhance self-efficacy specific to breastfeeding teaching and support.

Training materials included a branded kit with the following contents to support in-home breastfeeding counseling sessions with new mothers:

- Life-size newborn doll to demonstrate positioning;
- A skin-colored sock to prepare a breast model for demonstrating proper latching;
- A digital scale for weighing and assessing the growth of the infant;
- A nipple plunger to mitigate the problem of an inverted nipple;
- A Samsung Android tablet with the *BEST4Baby* app pre-loaded with wireless service and secured to allow for its sole use with the app.

Training content covered three key components: breastfeeding knowledge, counseling and communication skills, the use of the *BEST4Baby* app, and familiarity with overall PC responsibilities for each of the nine visits in the protocol for expectant or new mothers. Each session’s learning objectives, learning strategies, and evaluation process were predefined. The curriculum was designed to educate PCs for delivering timely information related to the mother’s stage of gestation (e.g., antepartum at 28 -32 weeks and 32-36 weeks) and the infant’s age (e.g., postpartum at 1-3 days, 15 days, 1 month, 2, 4 and 6 months) during nine home visits to provide support to the first-time mothers to practice exclusive breastfeeding. Tables 1 and 2 provide specific information about the curriculum content.

**Table 1:** Peer counsellor training content

**Session**	**Day 1**	**Time**
1	Registration; consent form signing; pre-test	30 min
2	Introduction Importance of breastfeeding and recommended practices Benefits of exclusive breastfeeding for 6 months Benefits for the mother Benefits for the child Benefits for the family Recommended practices How breastfeeding works	1 hour
3	Local breastfeeding situation and misconception	1 hour
4	Importance of Early initiation of EBF and pregnancy counselling	30 min
5	Demonstration of correct positioning and attachment Helping mothers hold the infant appropriately, with proper positioning Recommended rules of correct attachment Results of poor attachment How to produce sufficient milk How mother will understand that the infant is getting enough milk	30 min
6	Breastfeeding video and explanation	45 min
7	Expression of breast milk – Demonstration and practice	45 min
	**Day 2**	
8	Review of day 1 training	1 hour
9	Care of pregnant and lactating mothers and family planning Mother’s food and rest during pregnancy Iron tablets Check-up Delivery related information Family planning	30 min
10	Counselling skills with demonstration	1 hour
11	Counselling in pregnancy and role play (by the participants)	1 hour
12	Counselling in breastfeeding mother and role play	1 hour
13	Common breastfeeding difficulties and role play	2 hour
14	Review and clarification	90 min
15	Peer counsellor responsibilities and practical training sessions	1 hour
	**Day 3**	
16	App training with study protocol training	3 hour
17	Protocol review	3 hour
18	Post-test	30 min

**Table 2:** Peer counselor training curriculum

**Day Session**	**Content**	**Learning Objectives**	**Learning Activity**	**Evaluation**
Day 1 Session 1-4	Breastfeeding knowledge	Importance of breastfeeding and recommended practices Local breastfeeding situation and misconception Importance of early initiation of EBF Care of pregnant and lactating mothers and family planning	Brain storming Case based Interactive lectures	Pre- and post- questionnaire
Day 1 Session 4-7	Breastfeeding skills	Demonstration of correct positioning and attachment Assessment and observation of breastfeeding Expression of breast milk	Demonstration and practice with breastfeeding model and doll Breastfeeding video Assessment and observation of breastfeeding in real patients using the WHO Breastfeeding observation form	Pre- and post- questionnaire with LATCH^a^ criteria
Day 2 Session 8-9	Counseling skills during visits Breastfeeding mothers and mothers with breastfeeding problems	Understand counseling skills Learning and listening Confidence building and helping skills Using non-verbal and verbal technics to encourage mother to talk	Demonstration Interactive lecture /Brain storming case scenarios/role play of different scenarios Practice sessions -counseling sessions of real mothers in the postnatal ward Debriefing/ reflections	Pre- and post- Questionnaire
Day 2 Session 10	Peer counseling training	Understand responsibilities of peer counselors at each visit	Interactive lecture Role plays	
Day 3 Session 11	Use of Mhealth app	Understand usage and application of mobile app Study protocol training	Interactive lecture Demonstration and Practice	Peer Counselor usability scale for breastfeeding education app

^a^LATCH= “L” = how well the infant latches onto the breast; "A" = amount of audible swallowing noted; "T" = mother's nipple type; "C" = mother's level of comfort; "H" amount of help the mother needs to hold her infant to the breast.

The *BEST4Baby* app was designed to reinforce training by allowing PCs to use the device during and after the training sessions and in the field. A complete description of app content with screenshots is described in the work by Ma and colleagues [29]. The app's design included a step-by-step guide for each visit to cover relevant topics for that visit. Educational videos for the PCs to share with the mothers in the app included content on early initiation of breastfeeding, breastfeeding techniques, breastfeeding position and attachment, expression of breast milk, and myths of breastfeeding, The PCs were given the opportunity to use the app, including the process of logging in, scheduling visits, and practicing each visit. Time was allotted to practice the content of the training modules incorporated in the app.

## Methods

We describe here the pre- and post-evaluation of the initial training program for peer counselors in EBF and the use of the mHealth app conducted at the Research Unit of the Jawaharial Nehru Medical College in Belagavi, India. The Ethics Committee of the College approved the protocol for our work. All participants signed consent forms.

### Participants

Potential peer counselors were identified by staff from five primary health centers located in Belgavi, Karnataka, India and interviewed by research staff about their breastfeeding experience. Other inclusion criteria were (1) residing in the local community; (2) having breastfed within the past five years; (3) having at least 10 years of formal education; (4) having an available mobile phone; (5) being familiar with operating an Android phone; and (6) being able to read, write, and communicate in the local language.

### Assessment instruments

We evaluated participants in the breastfeeding training curriculum on knowledge, self-efficacy, skills in counseling, and usability of the mHealth application. These skill sets were evaluated employing a 30-item survey, adapted from the literature, to reflect curriculum content, validated in advance by twelve nursing and obstetric content experts who were members of the study’s Breastfeeding Advisory Panel. This panel included Karnataka MCH clinicians and academics, a Karnataka State Ministry of Health official, Reproductive and Child Health Officers, District Health Officers, and MCH advocacy group, La Leche League, and Baby Friendly Initiative representatives [30, 31]. The survey contained 20 knowledge items (e.g., colostrum is the first breast milk; breastfeeding helps in mother and child bonding); three counseling items (e.g., mother has a complaint about something that you, as a peer counselor, know little about. how do you respond?); two self-efficacy items (e.g., how confident are you in your ability to address mother’s concerns about breastfeeding?) and five observational skill items (e.g., is the infant correctly positioned?). Possible scores ranged from 0 to 20 on knowledge, 0-3 on counseling, 0-8 on self-efficacy, and 0-10 on skills subscales. At the end of the training, we also evaluated the app's usability using a modified System Usability Scale, a simple, widely available, validated 10-item survey based on a 5-point Likert scale and included two self-efficacy items [32]. Modification of this scale is commonly standardized by replacing "the system" with the name of the entity being evaluated, in this case, the app name. All instruments were translated into the local language of Kannada, back-translated for accuracy of content, and pilot tested with local women.

### Data collection

Data were collected from the PCs on paper forms at the start and completion of the training. Each PC was given a unique identifier to match their pre- and post-surveys.

### Data analysis

Data were entered into SAS 9.4 statistical program. [33] (SAS Institute, 2002-2012). In addition to descriptive statistics, matched pair T-tests were conducted to assess pre-and post- training changes.

### Results/evaluation of training

Fifty-six potential PCs were identified by staff from five primary health centers; 25 were selected who met the inclusion criteria. The 25 PCs had an average age of 29 years (SD 4.4; range 23-40), 88 % were married for an average of 11 years; 56 % had attended at least 11-16 years of school and 76 % had some form of formal work experience. All 25 PCs completed both pre and post-test assessments. Mean scores significantly improved from pre- to post-training on the breastfeeding knowledge (*p* < 0.0001), counseling (*p* = 0.0006), and skills (*p* = 0.0006) modules. Scores on the self-efficacy items did not change significantly. After the half-day training, scores on the Peer Counselor Usability Scale for the app were found to be highly usable, with the 25 PCs reporting an average score of 87.5 (SD ± 8.2; range 72.5-100).

## Conclusions

This study described and evaluated a new training curriculum for PCs to support EBF practices with effective counseling and a mHealth app. Results demonstrated that a brief 3-day training curriculum effectively trained regular mothers to become PCs with significant increases in the acquisition of PC’s knowledge, counseling, communication skills, and their ability to use the mHealth app.

Despite not having substantial prior knowledge and training in breastfeeding counseling, PCs obtained their skills through focused training, interactive pedagogy, and support to perform their counseling tasks effectively. Limited literature is available describing training curricula for PCs to promote breastfeeding within the local context. A comprehensive review of the PC training protocols and breastfeeding peer support programs revealed a wide divergence of content, type, tools, and aids, as well as the duration of the training and type of curriculum used; in several studies, few details were provided regarding the training process [34].

Effective training and support of PCs is a crucial step in ensuring the success of community-based peer support programs and is one of the proven effective strategies for increasing breastfeeding initiation and duration [35–37]. Our work highlights the need for a standardized training program and the development of training tools/aids, as well as the utilization of a mHealth app to support the implementation of a PC breastfeeding program. Similar to this study, all training must be adapted to local needs, provide up-to-date scientific information, and ensure skills development.

Peer Counselor training programs cited in earlier studies have used different modules and curricula for training (see examples in [36]). A systematic review and meta-analyses examined the effectiveness of community-based peer counseling on breastfeeding practices and found that most of the studies conducted in both developed and developing countries, employed the WHO/UNICEF breastfeeding manual/course for training [36]. Our curriculum had three key components: improving breastfeeding knowledge, counseling and communication skills, the use of a specially designed mHealth app, and elucidation of PC responsibilities during each of the nine visits to expectant or recently delivering mothers. While there exist varying modules for expanding breastfeeding knowledge and skills, we believe this to be among the first curricula which incorporate a specific mHealth app to enhance the impact of PC training on breastfeeding practices within a nine-visit breastfeeding intervention that provides “just-in-time” information to support mothers in their breastfeeding effort.

Limitations of this work include its one site, one time use. Future applications are planned, depending on funding availability. Strengths include the fact that, to our knowledge, a mHealth app for use by PCs to support lactation counseling has not been previously reported. Breastfeeding apps have been developed for use by mothers and support networks, including fathers [21, 22, 38]. The *BEST4Baby* mHealth app was specifically designed to provide a step-by-step guide for the PCs to conduct each of the nine visits and provide relevant content at each visit. Building the content for the mHealth app and training women with limited smartphone usage experience were among the greatest challenges in implementation.

Our work highlights the value of developing a structured, effective training curriculum for PCs to implement a community-based EBF initiative. Apart from facilitating the acquisition of knowledge and skills, the training also provided the PCs with the ability (self-efficacy) to use the mHealth app effectively to support the EBF program. The curriculum recognizes the importance of a mHealth app in implementing the program and ensuring adherence to the intervention protocol. The long-term impact of such PC training on EBF practices needs further study and ideally should be evaluated in diverse settings employing a larger group of PCs. The implementation fidelity of the training curriculum requires further validation.

## Data Availability

The datasets used and/or analysed during the current study are available from the corresponding author on reasonable request.

## Abbreviations

EBF: Exclusive breastfeeding
PCs: Peer counselors
WHO: World Health Organization
LMICs: Low-and middle-income countries
mHealth: Mobile health
apps: Applications
